# Silane Treatment as an Effective Way of Improving the Reinforcing Activity of Carbon Nanofibers in Nitrile Rubber Composites

**DOI:** 10.3390/ma13163481

**Published:** 2020-08-07

**Authors:** Bolesław Szadkowski, Anna Marzec, Przemysław Rybiński

**Affiliations:** 1 Institute of Polymer and Dye Technology, Faculty of Chemistry, Lodz University of Technology, Stefanowskiego 12/16, 90-924 Lodz, Poland; anna.marzec@p.lodz.pl; 2 Institute of Chemistry, Jan Kochanowski University, Żeromskiego 5, 25-369 Kielce, Poland; przemyslaw.rybinski@ujk.edu.pl

**Keywords:** NBR composites, carbon nanofibers, silanization, silane coupling agents, reinforcement

## Abstract

Two different silane treatment methods were used to improve the reinforcing activity of carbon nanofibers (CNF) in acrylonitrile-butadiene rubber (NBR) composites. The first method was chemical silanization with [3-(2-aminoethylamino)propyl]trimethoxysilane (APTS) in ethanol solution, preceded by oxidation of the CNF with H_2_SO_4_/HNO_3_. The second method was direct incorporation of silanes during preparation of the composites (in-situ silanization). Three different silane coupling agents were used: [3-(2-aminoethylamino)propyl]trimethoxysilane, (3-mercaptopropyl)trimethoxysilane (MPTS), and 3-ureidopropyltrimethoxysilane (UPTS). The NBR composites were prepared in an internal laboratory mixer, with increasing concentrations of pure or modified CNF. The crosslink density and flammability of the NBR-filled composites were analyzed, as well as their rheological and mechanical properties. The electrical conductivity of the composites was measured to assess the formation of CNF networks in the elastomer matrix. The morphology of the CNF was assessed by scanning electron microscopy (SEM). Both the dispersion of the CNF in the NBR matrix and the polymer-filler interactions were improved following silane modification, as shown in SEM images and by the Payne Effect. The composites were also found to have enhanced moduli, tensile strength, hardness, damping, and electrical conductivity. Chemical treatment proved to be more effective at improving the reinforcing effect of CNF in the elastomer matrix than in-situ silanization. The results of this study demonstrate the great potential of both in-situ and chemical silanization for the preparation of reinforced polymer composites filled with CNF.

## 1. Introduction

The discovery of new nanoscale materials with outstanding physico-chemical properties, such as nanotubes, nanofibers, and nanoclays, opens the way for the production of new multifunctional polymer composites with improved performance [1,2,3,4]. Nanoparticles are a highly effective filler material, enhancing the physical properties of polymer composites. Carbon nanofibers (CNF), characterized by large aspect ratios and nanometric sizes, hold promise for the fabrication of new reinforced polymer composites. The structure of CNF consists of a series of graphite basal planes stacked one on top of another at an angle of about 25° along the longitudinal direction of the fiber [5]. Generally, CNF have larger diameters than carbon nanotubes, which are among the most effective of all the nanofillers. The typical diameter of a CNF is in the range of 50–200 nm, with a length of up to 100 µm [6]. Although the physic-chemical properties of CNF are inferior to those of carbon nanotubes, they still have many advantages as reinforcing fillers, such as lower cost, higher purity, high stability, and relatively good mechanical strength. In addition, the active carbon atoms on the outer surfaces of the nanofibers are exposed, which effectively increases their chemical reactivity and thus their possibilities for its modification [7].

The unique structure and properties of CNF at a relatively low price make them an attractive nanofiller for different polymer composites. Epoxy composites filled with CNF developed by Gauthier et al. [8] showed significant reinforcement. Jimenez et al. [9] employed CNF as fillers for poly (methyl methacrylate) composites prepared in chaotic mixers, obtaining electrically conductive composites with very low concentrations of filler. Sanchez-Garcia et al. [10] compared the effects of CNF and carbon nanotubes on the properties of different biocomposites. Increasing the content of carbon nanotubes and CNF in the polymer matrix resulted in biocomposites with enhanced thermal stability, mechanical strength, and oxygen barrier stability. Other researchers have developed hybrid systems in which CNF were mixed with other materials to obtain polymer composites. Even at low concentrations of CNF, the composites showed improved performance [11,12,13]. Such studies suggest that CNF may be effectively applied to improve the performance of different polymeric mediums. However, the fact that CNF have an inert surface and a large aspect ratio hinders their uniform dispersion. The tendency of CNF to agglomerate also has a negative effect on the interfacial forces between CNF and the polymer matrix, resulting in poor reinforcement efficiency.

Attempts have been made to improve the dispersion of CNF in polymer matrixes using mechanical stirring [14], ultrasonication [15], surfactants [16], and ionic liquids [17]. Although all these methods may facilitate the uniform dispersion of CNF in a polymer matrix, their effects on the final properties of the composite are not always positive. Due to relatively weak physical interactions, the fillers are easily extracted from the matrix, resulting in limited load (or energy) transfer between the filler and the polymer matrix. Therefore, chemical treatment of the nanofiller surface is used to improve rubber-filler interfacial interactions via covalent bonding. Acid-treated, ozone-modified, and silanized CNF have each been reported to provide enhanced dispersion in various polymer composites, improving their mechanical and electrical properties [18,19,20,21]. Most studies have focused on improving the interfacial forces between modified CNF and epoxy resins or thermoplastic polymers. In comparison, elastomer-filled systems have received relatively little attention. In our previous work [17], we focused on the effects of different non-ionic surfactants and ionic liquids on the properties of nitrile rubber (NBR) filled with CNF. The results were quite satisfactory, as both the distribution of CNF in the NBR matrix and the properties of the final composites improved.

In the present study, we investigate the effects of silane treatment of CNF on the rheological, mechanical, and electrical properties of NBR composites, as well as on their flammability. The CNF were first oxidized in a mixture of concentrated H_2_SO_4_ and HNO_3_ and then silanized with aminosilane. The agglomeration tendency of the CNF following silane treatment was assessed by scanning electron microscopy (SEM) and dynamic light scattering. For the purposes of comparison, in-situ functionalization of CNF was also performed with various silane coupling agents: ([3-(2-aminoethylamino)propyl]trimethoxysilane, (3-mercaptopropyl)trimethoxysilane, and 3-ureidopropyltrimethoxysilane). Nitrile rubber composites filled with unmodified, oxidized, and silanized CNF were fabricated in a multistep-mixing procedure. We then examined the crosslink density, composite morphology, rheological behavior (Payne Effect), mechanical properties (tensile strength, tear resistance, damping, hardness), flammability, and electrical conductivity of the final NBR composites.

## 2. Materials and Methods

### 2.1. Raw Materials

Carbon nanofibers (CNF) (outer diameter 80–200 nm, length 0.5–20 μm) were purchased from Nanostructured and Amorphous Materials, Inc. (Katy, TX., USA). Three types of silane were purchased from Sigma-Aldrich Chemie GmbH (Schnelldorf, Germany): [3-(2-aminoethylamino)propyl]trimethoxysilane (APTS), (3-mercaptopropyl)trimethoxysilane (MPTS), and 3-ureidopropyltrimethoxysilane (UPTS). Acrylonitrile-butadiene rubber (NBR) (EUROPRENE^®^ N2845 GRN-acrylonitrile content 28%, Mooney viscosity (ML1 + 4) at 100 °C = 45) was supplied by Torimex-Chemicals Ltd. Sp. z o.o. (Lodz, Poland) and used as an elastomer matrix. The NBR compounds were crosslinked with sulfur (Siarkopol, Tarnobrzeg, Poland), mercaptobenzothiazole (Siarkopol, Tarnobrzeg, Poland), zinc oxide (Huta Będzin, Będzin, Poland), and stearic acid (POCH S.A., Gliwice, Poland). All of the materials were commercial grade.

### 2.2. Silanization of Carbon Nanofibers

**Figure 1:**
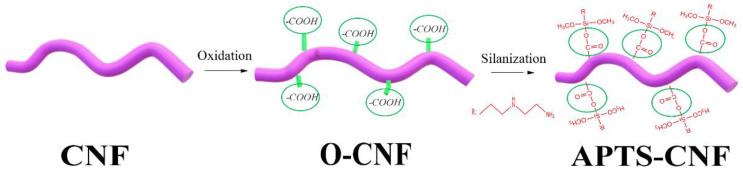
Schematic reaction of [3-(2-aminoethylamino)propyl]trimethoxysilane (APTS) silane coupling agent onto carbon nanofibers (CNF).

The chemical procedure for CNF silanization consisted of two steps. In the first step, as-received CNF were oxidized with a mixture of concentrated H_2_SO_4_ and HNO_3_ (volume ratio 3:1) in a water sonication bath at room temperature for 4 h. The oxidized carbon nanofibers (O-CNF) were then subjected to repeated filtration and washing with distilled water, until neutral pH was obtained. Afterwards, the obtained product was rinsed with acetone and further dried at 80 °C overnight. In the second step, silanization was performed by immersing a 5% concentration of acid-treated carbon nanofibers (oxidized CNF) in silane ethanol solution with the application of ultrasound (Hielscher Ultrasound Technology, UP400S, Teltow, Germany) for 2 h. The ethanol solution with [3-(2-aminoethylamino)propyl]trimethoxysilane (APTS) had been prepared beforehand. After the reaction, the solvent was evaporated using a rotary evaporator (Heidolph, Laborata4001 efficient, Schwabach, Germany). The obtained amino-silanized CNF (APTS-CNF) were dried in a vacuum oven at 60 °C for 24 h. A general scheme for the silane treatment of CNF is presented in Figure 1.

Mass silanization was also performed for comparison. The second method of mass functionalization was conducted during the compounding process (in-situ silanization). For this purpose, 3 phr (parts per hundred rubber) of each silane coupling agent was introduced with the other ingredients during preparation of the mixture in an internal mixer. Three different silanes (APTS, VMTS, and UPTS) were added to the elastomer blends immediately after the CNF.

### 2.3. Fabrication of Elastomer Composites

To prepare the NBR composites, all the ingredients except the curatives were prepared in an internal mixer (Brabender N50, Duisburg, Germany) in a multistep-mixing procedure [22]. In the first step, NBR rubber, zinc oxide, stearic acid, and CNF were blended at a temperature of 50 °C with a rotor speed of 50 rpm for 10 min. In the second step, the mixed compound was passed for mastication in a two-roll mill machine with roll dimensions: diameter (D) = 150 mm and length (L) = 300 mm. The curing agent was added and the rubber compound was mixed for 10 min. The average temperature of the rolls was 27–37 °C. Finally, the elastomer blends were removed from the two-roll mill and vulcanized on an electric heating press with a 1 mm-thick mold at 160 °C. The curing time was determined based on rheometric measurements. The formulations used to prepare the NBR/CNF vulcanizates were as follows:

- Neat NBR (Reference)
- NBR filled with 5 phr of CNF (5 CNF)
- NBR filled with 10 phr of CNF (10 CNF)
- NBR filled with 15 phr of CNF (15 CNF)
- NBR filled with 10 phr of oxidized CNF (10 O-CNF)
- NBR filled with 10 phr of APTS-silanized CNF (10 APTS-CNF)
- NBR filled with 10 phr of CNF modified in situ by APTS (10 CNF + APTS)
- NBR filled with 10 phr of CNF modified in situ by MPTS (10 CNF + MPTS)
- NBR filled with 10 phr of CNF modified in situ by UPTS (10 CNF + UPTS)

All the NBR composites, in addition, contained stearin (1 phr), zinc oxide (5 phr), mercaptobenzothiazole (2 phr), and sulfur (2 phr).

### 2.4. Characterization Techniques

The average size of the pure and modified CNF aggregates was determined based on the dynamic light scattering method (DLS) using a Zetasizer NanoS90 (Malvern Instruments, Malvern, UK) analyzer. The size of the particles was evaluated by dispersion in paraffin oil to simulate the conditions of the rubber matrix. Fourier transform infrared spectroscopy (FTIR) absorbance spectra were examined within the 4000–400 cm^−1^ range. The experiments were performed using a Thermo Scientific Nicolet 6700 FTIR spectrometer (Waltham, MA, USA) equipped with a diamond Smart Orbit ATR sampling accessory. The FTIR spectra were recorded in 64 scans at 4 cm^−1^ resolution. The thermal stability of the CNF was analyzed on a thermal analyzer (Jupiter STA 449F3, Netzsch Company, Selb, Germany), in a temperature range from 25 to 700 °C with a heating rate of 10 °C min^−1^. Differential scanning calorimetry (DSC) was performed on CNF samples of approximately 5 mg using a Netzsch DSC-204 apparatus (Netzsch Company, Selb, Germany), in a temperature range of 25–700 °C with a cooling and heating rate of 10 °C/min. The cure characteristics of the NBR compounds were evaluated at 160 °C using a D-RPA 3000 rheometer (MonTech, Buchen, Germany), according to PN-ISO 3417:1994. Based on the rheometric curves, the optimal vulcanization time (τ_90_), scorch time (τ_02_), minimum of torque moment (M_min_), and increment of torque (ΔM) were determined. The crosslink density of the NBR vulcanizates was calculated from measurements of equilibrium swelling in toluene. Four different samples of each vulcanizate were subjected to equilibrium swelling in toluene for 48 h at room temperature. The swollen samples were weighed, dried at a temperature of 50 °C to a constant weight, and reweighed after 48 h. Based on the results, the crosslink density of the vulcanizates was determined using Flory-Rehner’s Equation (1):

(1) $$\upsilon_{e}=-[ln\left(1-V_{r}\right)+V_{r}+µ\cdot V_{r}^{2}]/[V_{0}\cdot \;\left(V_{r}\left(1/3\right)-\left(V_{r}/2\right)\right],$$

where *υ_e_* is the crosslink density, *V_r_* is the volume fraction of the elastomer in swollen gel, *V*_0_ is the molar volume of the solvent (mol/cm^3^), and µ is the Huggins parameter.

To determine the content of non-covalent crosslinks in the elastomer, network samples were swollen in toluene in a desiccator with saturated chloroform vapor. The flammability of the NBR composites was evaluated based on microscale combustion calorimetry (MCC), using an MCC micro-calorimeter (Fire Testing Technology Limited, East Grinstead, UK). The samples were heated using a linear temperature program. The temperature of the pyrolyzer was 750 °C, and the temperature of the combustor was 900 °C. The physical properties of the composites, such as tensile strength at break (T_S_) and elongation at break (E_B_), were measured according to the PN-ISO 37:2007 standard using a Zwick tensile tester (Zwick, Ulm, Germany), model 1435, for 1 mm dumbbell-shaped samples. Average results were calculated for five samples of each composite. The hardness of the composites was assessed following the ISO 868 standard using a Shore Type A Durometer (Zwick, Ulm, Germany). The results for five points chosen randomly on each sample were averaged. The relative damping of the NBR vulcanizates was measured according to the PN-C-04289 standard, using a universal machine (Zwick, Ulm, Germany) at room temperature. Measurements were performed using disc-shaped samples with a diameter of 35 mm and height of 17.5 mm. Each sample was subjected to cyclic stress from 0 to 0.7 MPa in a five cycle loading–unloading program. Based on the obtained hysteresis loops, the relative damping values were determined according to Equation (2):

(2) $$T_{\tau w}=\frac{∆W_{i}}{W_{ibel}}\times 100,$$

where *T*_τw_ (%) is the relative damping, Δ*W_i_* is the difference between the compression work and the work of reducing the compressive stresses, and *W_ibel_* is the compression work. The morphology of the CNF and NBR/CNF composites was evaluated using SEM with a LEO 1530 Gemini scanning electron microscope (Zeiss/LEO, Oberkochen, Germany). The samples were broken in liquid nitrogen and sputter-coated with carbon under a high vacuum before examination. Optical microphotographs of the CNF were taken using a Digital Microscope Controller on a VHX-7000 Series microscope (Keyence, Japan). Dynamic measurements of viscoelastic properties were taken using an oscillation Ares G2 rotational rheometer (TA Instruments, New Castle, NY, USA). Two parallel plates of 25 mm in diameter were used. Rheological tests were performed under the following conditions: force 5 N, temperature 25 °C, sample deformation rate 10 rad/s, and stress from 0.1% to 150%. The Payne effect of the composites was determined based on Equation (3) [23]:

(3) $$∆G^{\prime }={G^{\prime }}_{max}\left(lim10^{-1}\right)-{G^{\prime }}_{min}\left(\infty \right),$$

where *G′_min_*(*lim* 10^−1^) is the composite storage modulus determined under deformation of 10^−1^% and *G′_max_*(∞) is the composite storage modulus determined under the max deformation. The resistivity of the NBR composites was measured using an insulation MIC-1000 resistance meter (SONEL S.A., Poland) coupled to EP-1 measuring electrodes. Based on the resistivity results, the surface electrical conductivity of the samples was determined using Equation (4):

(4) $$\sigma_{S}=\frac{1}{\rho_{s}},$$

where $\sigma_{S}$ is surface conductivity (S) and $\rho_{s}$ is surface resistivity (Ω) calculated from the formula:

(5) $$\rho_{s}=81.68\cdot R_{s},$$

where *R_S_* is measured surface resistivity (Ω).

## 3. Results

### 3.1. Characterization of the Carbon Nanofibers

**Figure 2:**
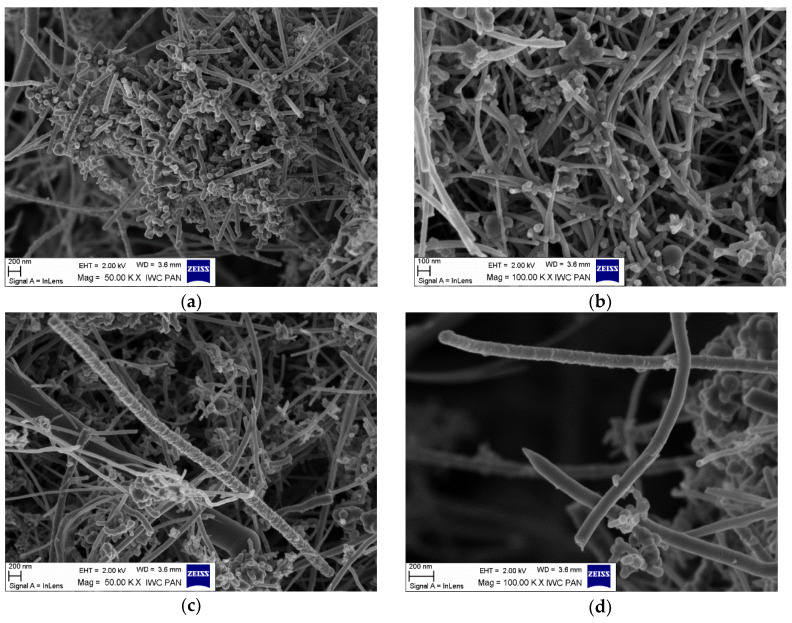
Scanning electron microscopy (SEM) images: (**a**,**b**) untreated carbon nanofibers, and (**c**,**d**) carbon nanofibers modified with APTS at 50 and 100 KX magnification.

**Figure 3:**
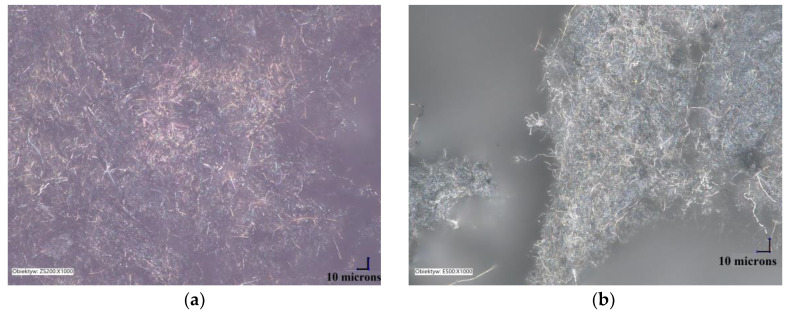
Optical microscopy images: (**a**) raw carbon nanofibers, and (**b**) APTS-modified carbon nanofibers.

The morphology of the unmodified and silane-modified CNF was characterized by SEM and optical microscopy. The images obtained are presented in Figure 2 and Figure 3. Carbon nanofibers were identified with nanometer-scale widths of 50–100 nm. The diameters of the original CNF were within in the range of a few microns, which is in line with the values provided by the manufacturer. From Figure 2a,b, it can be seen that the CNF have a fibrous structure with a smooth surface and exhibit a high tendency to form complex entanglements, obviously due to their high aspect ratio. This tendency to agglomerate could have a strong negative influence on the mechanical strength of elastomer composites, since large clusters of nanofiller can act as a stress concentrator in polymer matrixes, causing local weakening of the composites. Therefore, to enhance interfacial compatibility between the CNF and the nitrile rubber matrix, the CNF were first oxidized and then silanized with [3-(2-aminoethylamino)propyl]trimethoxysilane. Figure 2c,d shows that the silanization conditions did not affect the morphology of the nanofibers (especially their length), so the process is nondestructive to the CNF structure. However, silanization contributed to the appearance of an additional thin layer on the CNF surface, which can be attributed to the coating formed by the silane. It is possible that the APTS silane agent applied underwent hydrolysis under the studied conditions. Thus, the single spherical particles visible on the SEM images may be the result of a silane condensation reaction [24]. Silane modification successfully changed the surface properties of the CNF, while the fiber dimensions remained unchanged. It was also observed that the modified CNF formed significantly smaller entanglements and agglomerates compared to the untreated CNF.

**Figure 4:**
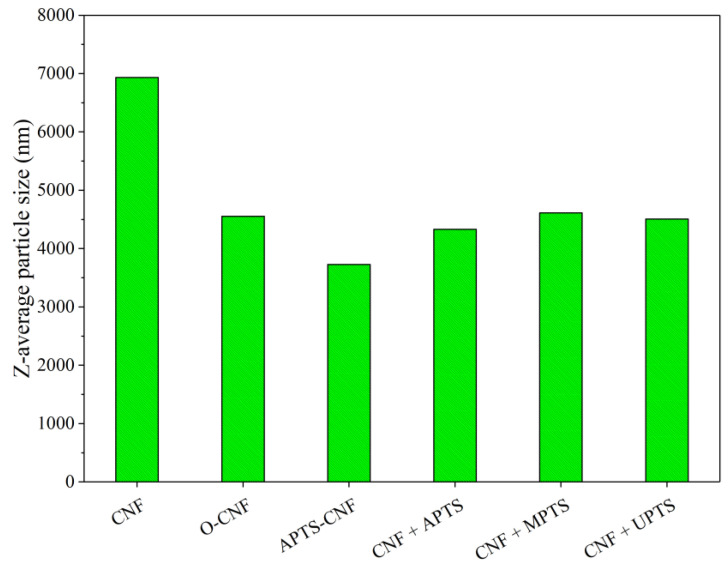
Aggregate sizes for CNF particles modified with different silane coupling agents.

Very useful information concerning the tendency of the CNF particles to agglomerate depending upon surface modification was obtained using dynamic light scattering (DLS) size analysis. Figure 4 shows the average sizes of the agglomerates formed by the CNF modified with different silane coupling agents in paraffin oil (to imitate the elastomer medium). The DLS results clearly show that the raw CNF exhibited a very high tendency to agglomerate, since the Z-average sizes for this sample reached about 7000 nm. However, chemical treatment of the CNF contributed to reduce the sizes of the agglomerates by approximately 2500–3000 nm. The most pronounced reduction was observed for the APTS-CNF sample, as the average agglomerate size decreased by up to 3600 nm. Taking into account the DLS results, it may be assumed that silanization inhibits the agglomeration of CNF and can improve their compatibility and dispersion in the elastomer matrix.

**Figure 5:**
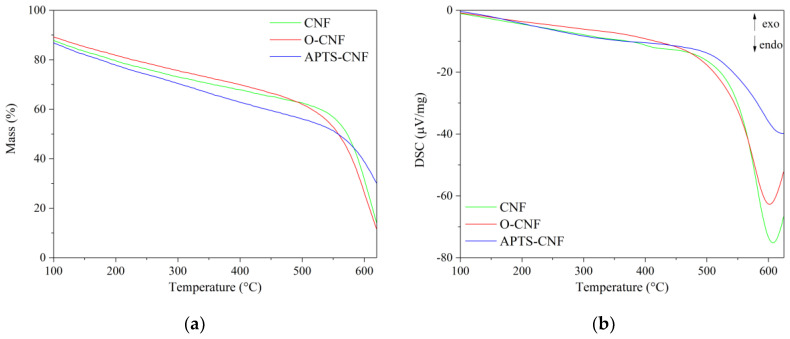
(**a**) Thermogravimetric and (**b**) differential scanning calorimetry (DSC) curves for raw, oxidized, and APTS-modified carbon nanofibers.

Thermogravimetric analysis (TGA) and differential scanning calorimetry (DSC) revealed that the CNF had very high stability at elevated temperatures. The TGA and DSC curves for both raw and modified CNF are presented in Figure 5. As can be seen in Figure 5a, the CNF were thermally stable materials, with a maximum decomposition temperature of T_RMAX_ = 560 °C. In addition, the silanization of CNF with aminosilane contributed to shift the maximum decomposition temperature towards higher temperatures by approximately 26 °C, reaching T_RMAX_ = 586 °C. The T_50%_ weight loss temperatures for the unmodified CNF, oxidized CNF, and APTS-modified CNF were 573, 558, and 560 °C, respectively. As shown by the DSC curves (Figure 5b), the endothermic transformations associated with CNF, O-CNF, and APTS-CNF decomposition were recorded at 605, 600, and 625 °C, respectively. All the studied CNF exhibited extremely high thermal stability, which was also reflected in the improved flame resistance of the elastomer composites filled with CNF.

### 3.2. Rheometric Studies

**Figure 6:**
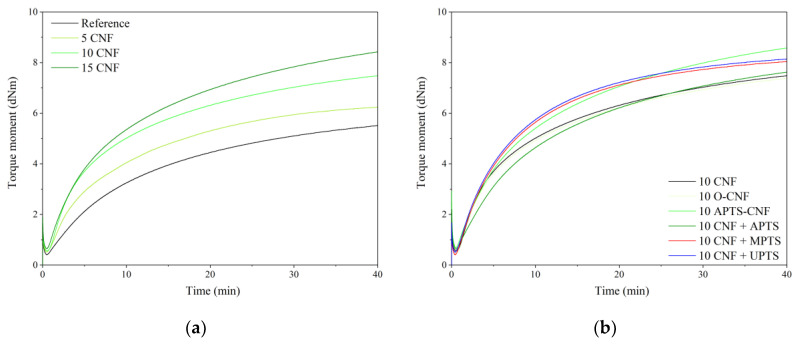
Cure curves for acrylonitrile-butadiene rubber (NBR) compounds filled with (**a**) different loadings of CNF, and (**b**) CNF modified with different silanes.

The vulcanization behavior of the NBR compounds containing various concentrations of modified and unmodified CNF was studied using rheometric measurements. The curing curves are shown in Figure 6 and the rheometric parameters are presented in Table 1. In Figure 6, long marching curves can be observed for all the studied compounds. The marching trend of the NBR curing curves seems to be characteristic for nitrile rubber composites cured with a sulfur-based crosslinking system, as a similar phenomenon was observed also in our previous works on NBR [25,26,27]. In Figure 6 and Table 1, a remarkable increase can be seen in the torque for the NBR/CNF blends, which is due to the immobilization of polymer chains in the rigid CNF region. Once the CNF was loaded, the M_min_ and ΔM increased consistently with increasing concentrations of filler. This can be explained by the enhanced viscosity of the samples, which is induced mainly by the network structure of CNF that formed within the polymer matrix. The improved ΔM may indirectly indicate enhanced crosslink density after the incorporation of CNF. The application of CNF also resulted in the reduction of scorch time and optimal vulcanization time.

Carbon nanofibers are known to possess a high surface-to-volume ratio and a narrow, hollow core, which is encompassed by cylindrical graphene layers of increasing diameter. This can facilitate the favorable entrapment of cure functionalities, resulting in earlier onset of curing and reduced scorch safety times in comparison to unfilled rubber [28,29]. After silane modification of the CNF, the curing times remained almost unchanged with respect to the 10 CNF sample. However, it is interesting to note that ΔM increased for NBR compounds filled with silanized CNF compared to the 10 CNF sample. The most pronounced improvement was observed for chemically modified CNF. The ΔM parameter was enhanced for the 10 APTS-CNF sample by 0.82 dNm compared to the 10 CNF composite. The improved polymer-filler interactions after modification of CNF with silane agents probably contributed to the marked improvement in crosslink density and stiffness of this compound. The minimum torque (M_min_) decreased for samples containing silane-modified CNF compared to the composite with raw CNF. Generally, the minimum torque moment corresponded to filler-filler inter agglomeration in the elastomer system [30]. The lower the M_min_ value, the weaker the filler-filler interaction, resulting in lower viscosity of the compound. These results suggest that filler-filler interactions were reduced by the application of silane coupling agents.

### 3.3. Crosslink Density Measurement

**Figure 7:**
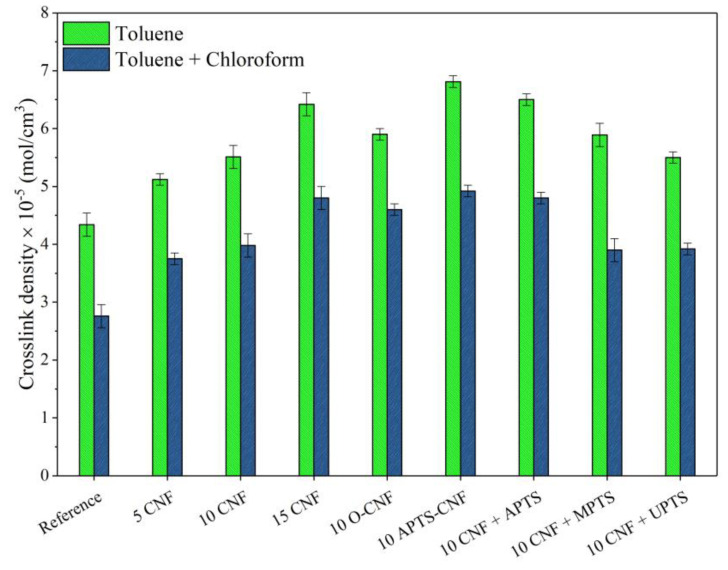
Crosslink density values of the NBR vulcanizates filled with unmodified and chemically treated carbon nanofibers.

The crosslink density of the cured NBR elastomer was determined from swelling measurements and crosslink density values, as calculated using the Flory-Rehner equation (Figure 7). Adding a low concentration of the reinforcing nanofiller to the elastomer matrix was sufficient to increase the crosslink density of the NBR vulcanizates. As the filler content increases, there was also a clear increase in the total crosslink density. The crosslink density value improved gradually from 4.2 mol/cm^3^ for neat NBR up to 5.1, 5.6, and 6.6 mol/cm^3^ for 5, 10, and 15 phr of CNF, respectively. This is in agreement with the results presented in other works on reinforcing nanofillers, such as carbon black, carbon nanotubes, or nanofibers in nitrile rubber composites [25,26,31,32]. At the same CNF loading (10 phr), the vulcanizates filled with silanized CNF showed considerably higher values for crosslink density compared to vulcanizate filled with untreated CNF, since the presence of silanes promoted CNF-rubber bonding and improved the dispersion of the CNF in nitrile rubber matrix. As predicted from rheometric measurements, CNF modified with APTS had the most pronounced effect on this parameter. Chemical modification in ethanol (APTS-CNF) proved to have a slightly greater impact on crosslink density compared to the in-situ silanization process (CNF + APTS). This can be explained by more effective adsorption of silane on the outer surface of the CNF, due to increased numbers of hydroxyl and/or carboxylic moieties after the oxidation process. As a further consequence, the chemical modification of CNF facilitated interactions between the silane on the CNF surface and the rubber macromolecules, as well as more uniform dispersion of the CNF in the NBR matrix. Nevertheless, the crosslink density values obtained for vulcanizates filled with 10 phr of CNF treated with silane coupling agents may be ordered as follows: 10 CNF < 10 CNF + UPTS < 10 O-CNF < 10 CNF + MPTS < 10 CNF + APTS < 10 APTS-CNF. It was also found that non-covalent bonds formed in the network structure and then broke under the influence of chloroform vapors, as revealed by the decrease in the crosslink density measured on the basis of equilibrium swelling in toluene and chloroform vapors. The highest content of ionic crosslinks was observed for the sample containing 10 phr of APTS-CNF, which confirms that the application of aminosilane resulted in improved interactions between the filler and polymer matrix.

### 3.4. FTIR Analysis and Morphology of the Elastomer Composites

**Figure 8:**
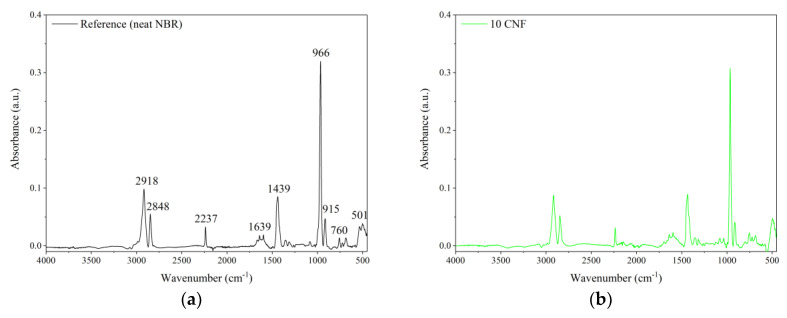

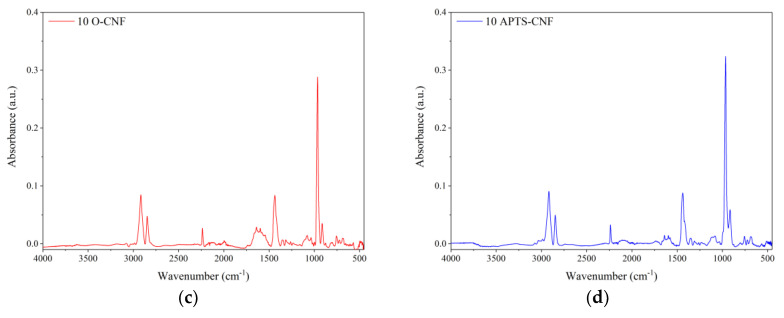
Fourier transform infrared spectroscopy (FTIR) spectra of the studied composites: (**a**) unfilled NBR, (**b**) NBR filled with 10 phr of CNF, (**c**) NBR filled with 10 phr of oxidized (O)-CNF, and (**d**) NBR filled with 10 phr of APTS-CNF.

The FTIR spectra of the unfilled NBR and nitrile rubber composites containing 10 phr of raw and modified CNF are shown in Figure 8. Generally, the FTIR spectra show characteristic peaks for NBR elastomer, such as 2918 cm^−1^ and 2848 cm^−1^, which can be attributed to υ(CH_3_–, –CH_2_, >CH–) asymmetric stretching vibration, as well as 2237 cm^−1^, which is characteristic of υ(CN–) stretching vibration. The peaks at 1639 cm^−1^ and 1439 cm^−1^ may be assigned to υ(–CH=CH–) symmetric stretching vibration and δ(–CH_2_–) bending vibration, respectively. The strong and very narrow peak located at 966 cm^−1^ as well as the peak at 760 cm^−1^ may be attributed to CH wagging of trans and to the cis –CH=CH unit of the butadiene segment [33,34]. The relative positions of the main characteristic bands for various functional groups in the NBR-filled composites are similar to those for the reference outcomes. However, some slight alterations in the band region of 1800–600 cm^−1^ occurred after application of CNF, especially oxidized CNF. This region is commonly ascribed to the presence of C=O functional groups on the material surface [35]. Therefore, the increased intensity of the peak at 1639 cm^−1^ observed on the 10 O-CNF sample spectrum may suggest the presence of more carbonyl groups after the oxidation of CNF.

**Figure 9:**
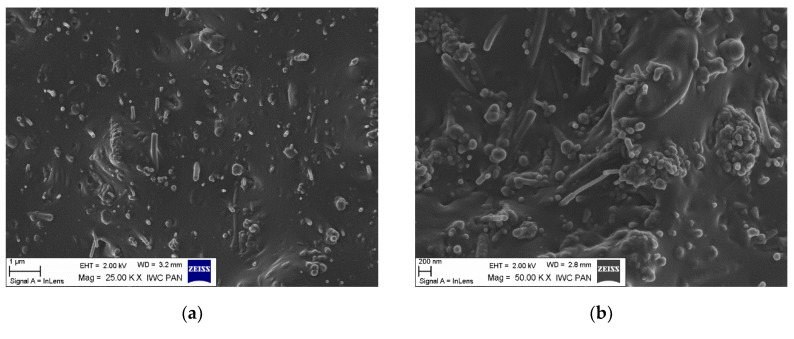

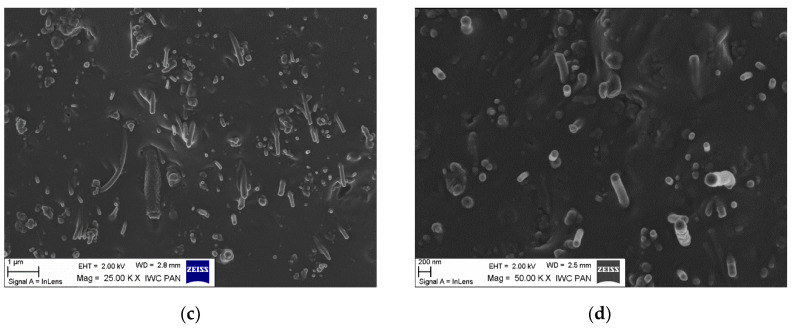
Scanning electron microscopy (SEM) images of NBR composites filled with (**a**,**b**) 10 phr of CNF, and (**c**,**d**) 10 phr of APTS-CNF.

Achieving uniform distribution of nanofillers is vital for manufacturing final composites with improved properties. The microstructure of the NBR composites filled with CNF was analyzed based on SEM. Figure 9 presents SEM micrographs of fractured surfaces of cured NBR composites filled with 10 phr of unmodified CNF and APTS-CNF. At the micron scale, numerous CNF nanoparticles can be observed in the rubber matrix, which are clearly characterized by a fibrous structure hundreds of nanometers in length. Owing to the large aspect ratio and longitudinal fibrous structure of CNF, they have a high tendency to form complex entanglements and thus agglomerations, as confirmed by DLS measurements. The formation of agglomerates by CNF nanoparticles was observed clearly in the elastomer matrix. The SEM images of NBR composites filled with unmodified CNF (Figure 9a,b) show many large and massive clusters of CNF in the NBR matrix, due to the relatively weak interfacial interactions between the nanofiller and the elastomer. This is one of the most important drawbacks of using nanofillers in elastomer technology. Meanwhile, as shown in Figure 9c,d, many individual fibrous particles were observed uniformly distributed in the composite filled with APTS-CNF, indicating better filler-polymer compatibility and more homogenous dispersion of the nanofiller in the rubber matrix after modification. Therefore, local stress may be more efficiently transferred into silanized CNF, resulting in higher mechanical strength. Nie et al. [20] suggested that silanization of CNF produces better dispersion and stronger adhesion between CNF and the polymer matrix because of improved interfacial forces. This is why silanization may have an important role in improving the reinforcing efficiency of CNF in elastomer composites.

### 3.5. Mechanical Properties

**Figure 10:**
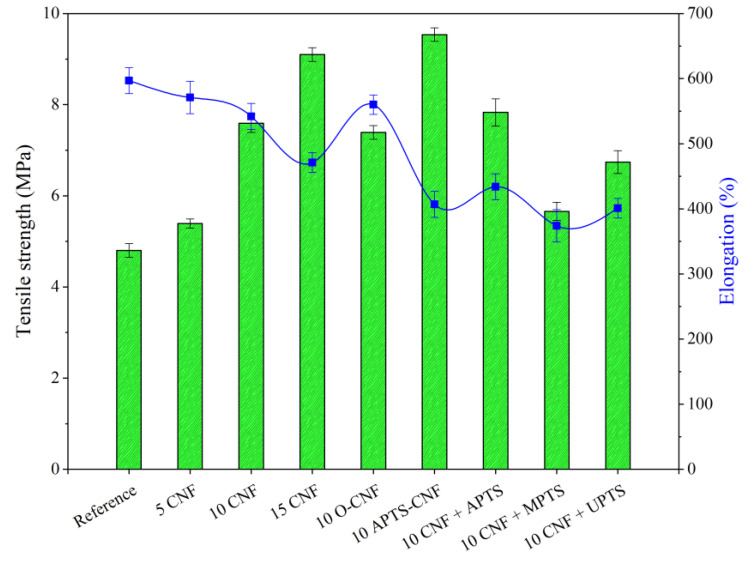
Tensile strength test results obtained for NBR composites with modified carbon nanofibers.

**Figure 11:**
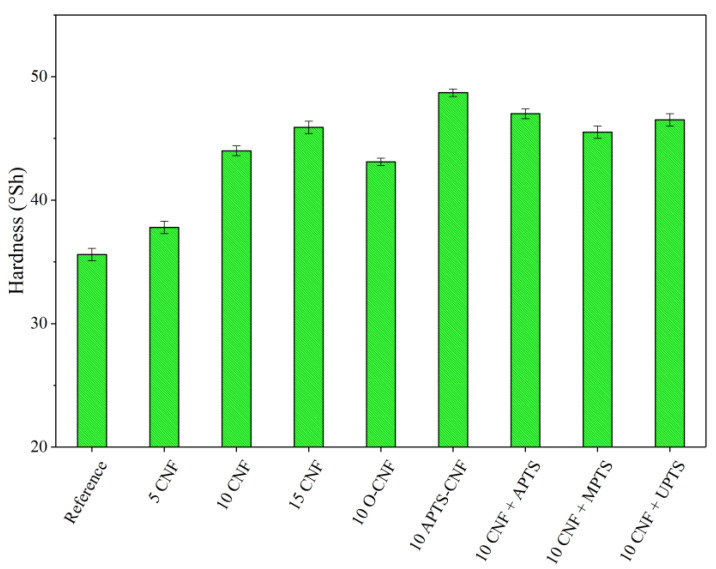
Hardness parameters obtained for NBR composites with modified carbon nanofibers.

**Figure 12:**
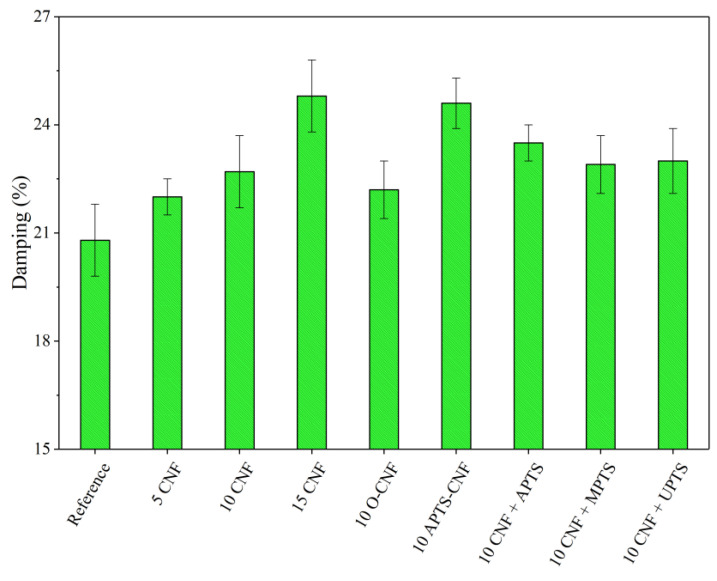
Relative damping parameters obtained for NBR composites with modified carbon nanofibers.

To take full advantage of the superior reinforcing ability of CNF, excellent adhesion must be achieved between the polymer matrix and filler. Therefore, we focused on improving polymer-filler compatibility via silane treatment of the CNF. We analyzed the effect of this modification on the mechanical properties of the NBR/CNF composites using the tensile strength test, as well as damping and hardness analysis. The results are presented in Figure 10, Figure 11 and Figure 12. For the NBR filled with 5 phr of CNF, increases were observed of about 17% in tensile strength, 8% in hardness, and 7% in damping. This suggests that the composite was stronger than the neat NBR (Reference), even after the incorporation of only a small amount of CNF. As expected, further increases in the proportion of CNF in the composites resulted in a gradual increase in the values of these parameters. This is typical for reinforcing carbon nanofillers that strongly affect the mechanical performance of elastomers [36]. The elongation at break, which is an indicator of material flexibility, followed a decreasing trend with increasing loads of CNF. This is typical for polymer composites filled with rigid filler particles, which stiffen the composite material. Unexpectedly, the opposite effect was observed for O-CNF. The tensile strength value for this sample was slightly lower than that for the composite with 10 phr of raw CNF. The vulcanizate was significantly stiffer, as evidenced by lower elongation at break values, which resulted in more rapid breaking of the samples.

Silanization of the CNF caused satisfactory reinforcement of the nitrile rubber matrix. The reinforcing effect of the modified composites can be ordered as follows: 10 CNF < 10 MPTS + CNF < 10 UPTS + CNF < 10 APTS + CNF < 10 APTS-CNF. This agrees with the torque differences and crosslink density measurements, in that the addition of silanized CNF improved CNF-rubber interactions and dispersion in the NBR matrix. Other authors have noted the positive effect of filler surface modification with silane coupling agents on the mechanical properties of elastomer composites [37,38]. Improved reinforcement effects have been reported for polymer composites filled with silane-modified fillers such as silica, clays, and graphene oxides [39,40,41]. In our study, the silanization of CNF with APTS led to strong chemical bonding, which promoted compatibility between the fibers and rubber while also increasing the interfacial adhesion between the two phases, resulting in the best mechanical performance of the final NBR composites. The tensile strength of the composites filled with 10 CNF + APTS and 10 APTS-CNF increased considerably, from 6.9 MPa (10 CNF) up to 7.9 and 9.5 MPa, respectively. It can be concluded that the chemical modification of CNF with APTS silane in ethanol (10 APTS-CNF sample) affected the reinforcing potential of the CNF particularly strongly, since the tensile strength value exceeded even that obtained for 15 CNF (9.1 MPa). Polar aminosilane facilitated better dispersion of CNF in the elastomer matrix, while amine functional groups may have reacted with the nitrile rubber during the curing process, strengthening the effective mechanical load transfer from the rubber matrix to the nanofiller. The positive effect of silane treatment of the CNF on the mechanical properties of the nitrile rubber composites was also confirmed by improved hardness and damping properties. As the CNF content was increased, these values also rose, in an almost linear way. Again, the reinforcement of the vulcanizates was significantly more effective after the application of silanized CNF.

### 3.6. Payne Effect

**Figure 13:**
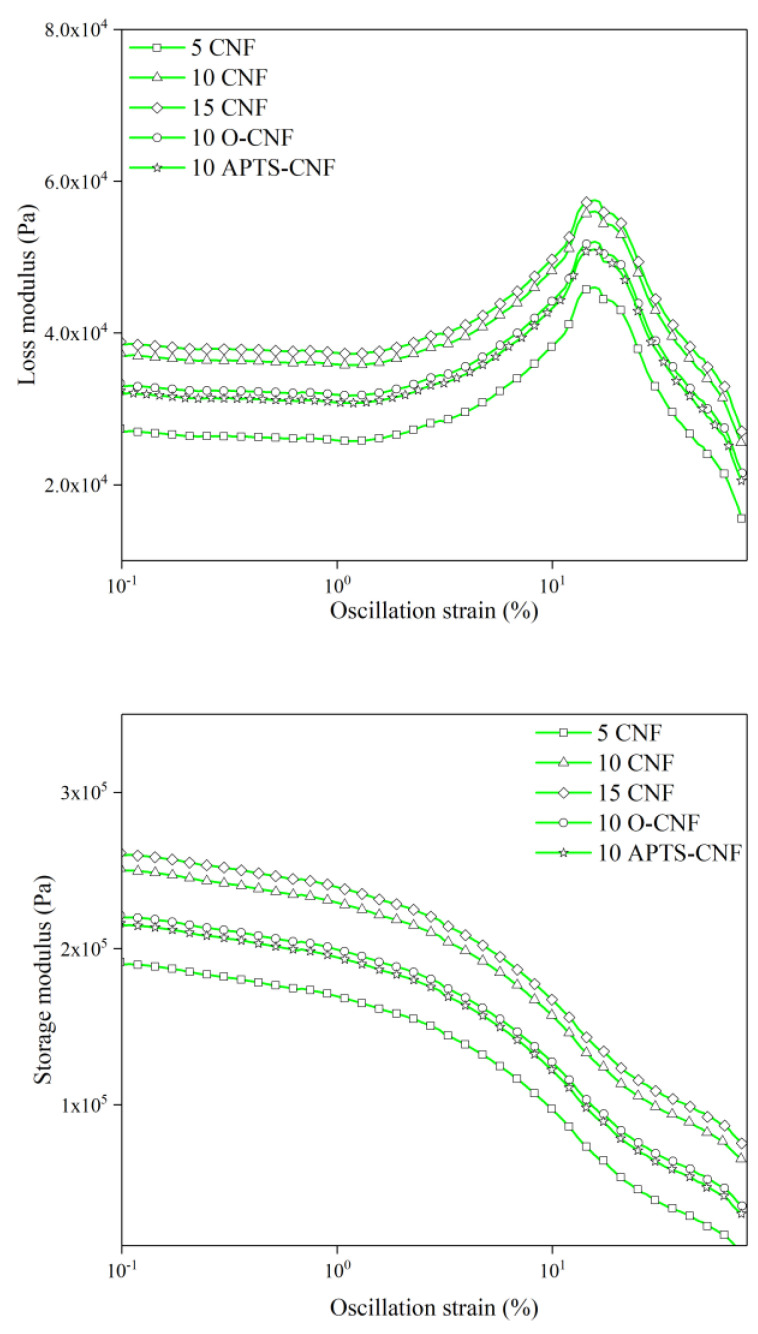
Dynamic moduli of NBR filled with carbon nanofibers as a function of oscillation strain.

According to Payne and Wang [42,43], the incorporation of an active filler into an elastomer matrix results in an increase in the elastic modulus, due to the formation of the secondary structure of the filler in the elastomer. The viscoelastic properties of elastomer composites filled with nanofillers are strongly dependent on the size of the dispersed filler phase and its distribution throughout the rubber matrix. These effects are further magnified if additional interactions (e.g., polar, chemical, van der Waals interactions) occur at the polymer-filler interface. When the deformation amplitude increases during dynamic measurements, the moduli of the filled composites decreases due to the breaking of the interactions between the filler aggregates (van der Waals and hydrogen bonding). This phenomenon is known as the Payne Effect. It can be assumed that silanization of CNF may improve their compatibility with NBR, and consequently improve the polymer-filler interactions. Since the Payne effect depends mainly on the hydrodynamic effect as well as on filler-filler interactions, we anticipated that the modified samples would show lower Payne effect values. In order to gain investigate the filler-filler interactions and the compatibility between the modified CNF and the elastomer matrix, the dynamic modulus was analyzed as a function of the oscillation strain. The dynamic measurements results are presented in Figure 13 and Table 2. As can be observed from Figure 13, the storage modulus (G′) initially remains constant for all samples at an oscillation strain below 1% and then falls sharply due to the destruction of the secondary structure of the filler in the polymer matrix. Meanwhile, the loss modulus (G″) passes a maximum at a critical strain of about 10% and then decreases.

The results of dynamic modulus measurements reveal that the CNF formed a strong complex structure in the NBR matrix, since increasing the content of CNF in the NBR elastomer led to higher values for the G′ and G” parameters, especially in the small deformation range. This is due to the fact that inter-aggregate distances become smaller with greater filler loading, and therefore the probability of the formation of a filler network increases. Similar observations have been reported by Georgousis et al. [44], Bokobza et al. [45], and Fu et al. [46] for elastomer composites filled with carbon black, carbon nanotubes, and silica, respectively. Importantly, interactions between the nitrile rubber and the CNF surface were improved markedly by the silane coupling agents, as shown in Figure 13 and Table 2. The NBR composites filled with untreated CNF had a larger magnitude of the Payne Effect, meaning more powerful filler-filler interactions and correspondingly poor filler-rubber interactions [47]. Therefore, it can be stated that the silanization of CNF contributed to better compatibility between the CNF and rubber, as well as improved dispersion of the nanofiller in the elastomer matrix. This is in agreement with results reported by other researchers [48,49,50], which show that the Payne Effect may be reduced noticeably following the silane treatment of various fillers. The NBR composites filled with CNF modified with aminosilane reached the highest ΔG′ in comparison to the other silanized samples. Amino groups can form hydrogen bonds with other amino and hydroxyl groups even after modification, thus the 10 APTS-CNF compound showed a much higher ΔG′.

### 3.7. Electrical Properties

**Figure 14:**
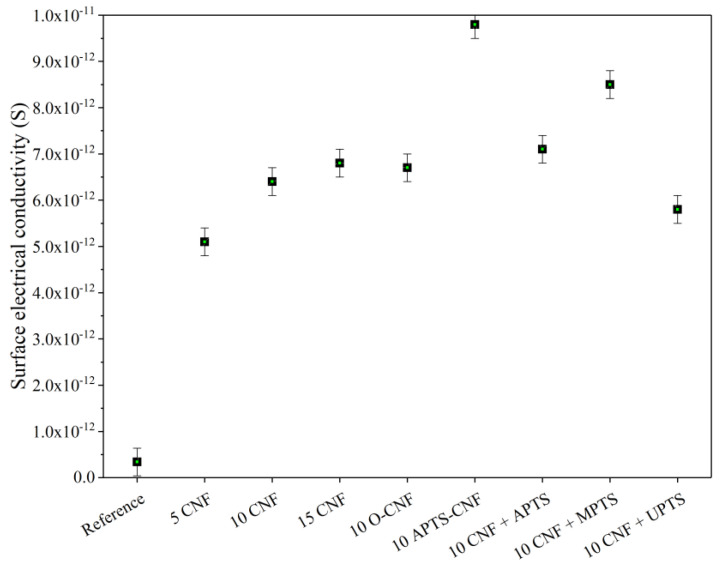
Surface electrical conductivity of NBR composites filled with modified carbon nanofibers.

Electrical conductivity measurements can be used as an effective indicator of the quality of filler dispersion in an elastomer composite. If a continuous filler network of conductive filler particles forms at a particular concentration, a sharp increase can be observed in the electrical conductivity of the polymer composite. This phenomenon is known as the percolation threshold. As shown in Figure 14, there was a sharp increase in conductivity when the CNF concentration in the composite reached 5 phr. This sudden rise in electrical conductivity was an effect of the formation of a continuous network of CNF in the elastomer matrix. Further increases in the CNF concentration caused only marginal improvements in electrical conductivity, since the percolation threshold had already been achieved. From Figure 14, it may also be noticed that the conductivity of the NBR composites filled with 10 phr of CNF was improved by the addition of silane coupling agents, except in the case of UPTS. Nakaramontri et al. [50] reported that silane coupling agent may improve the dispersion of carbon fillers, due to enhanced chemical interactions which result in natural rubber composites with superior electrical properties. In our study, the most pronounced increase in conductivity was observed for the 10 APTS-CNF sample, which confirmed our previous assumptions regarding the improved dispersion and polymer-filler compatibility of this sample in particular. The silanization of CNF facilitated electron tunneling at the particle linkages, and therefore, the electrical conductivity of the vulcanizate was greatly enhanced.

### 3.8. Flammability

**Figure 15:**
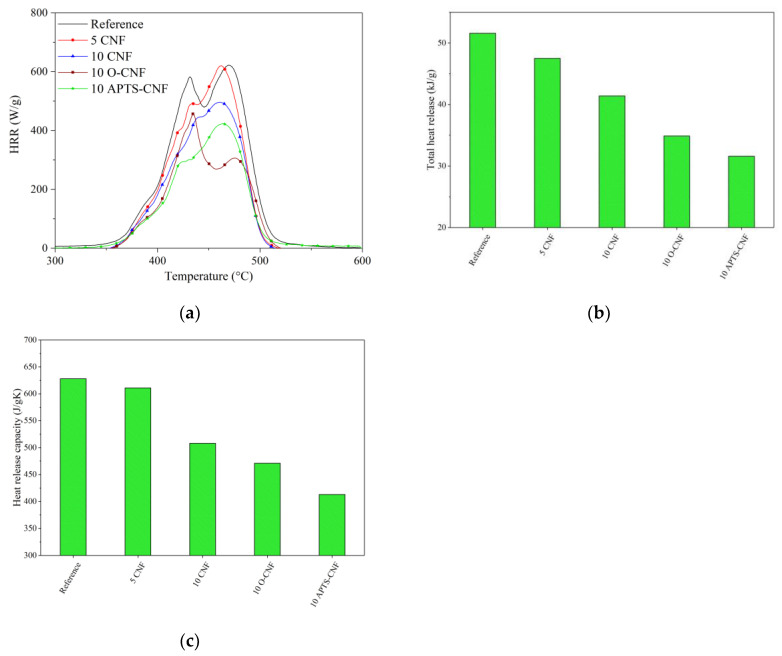
Results of microscale combustion calorimetry measurements: (**a**) heat release rate, (**b**) total heat release, (**c**) heat release capacity.

The flammability of the NBR composites filled with raw, oxidized, and silanized CNF was analyzed using a microscale combustion calorimeter (MCC). The MCC test is a verified method for measuring the static (heat release capacity (HRC), total heat release (THR)) as well as dynamic (heat release rate (HRR)) combustion parameters of different polymeric composites. The flammability parameters obtained using the MCC test are presented in Figure 15.

As shown in Figure 15, the CNF clearly decreased the fire hazard of the crosslinked NBR composites. This was reflected in a significant reduction in the flammability parameters following the incorporation of CNF into the NBR matrix. When lower concentrations of CNF were introduced, such as 5 and 10 phr, the total heat release (THR) parameter reduced sharply from 52 kJ/g for neat NBR to 47 kJ/g for the 5 CNF sample and 41 kJ/g for the 10 CNF sample. As expected, an analogous trend was observed for the other combustion parameters after the introduction of CNF to the elastomer matrix. According to the literature [51,52,53,54], the predominant mechanism whereby the flammability of polymer composites filled with nanofillers is reduced involves the formation of a network structure of nanoparticles in the polymer matrix, which slows down polymer mass loss (fuel release) under fire conditions. This effect probably also explains the reduction in the flammability of the NBR composites used in our study following the addition of CNF. However, the NBR systems filled with APTS-CNF showed a more significant decrease in flammability, with reductions in HRR, THR, and HRC parameters of more than 20%. The decrease in flammability of the NBR-filled composites after the application of silanized CNF may be attributed to the more uniform dispersion and better compatibility of the APTS-CNF in the NBR system, which resulted from improved polymer-filler interactions following the silanization process. The silane treatment of CNF resulted in the more homogeneous boundary layers formed by CNF nanoparticles. The boundary layer decreased the mass and energy transfer between sample and flame and in the consequence, decreased the flammability of the composite.

## 4. Conclusions

In this work, three different silane coupling agents: ([3-(2-aminoethylamino)propyl]trimethoxysilane, (3-mercaptopropyl)trimethoxysilane, and 3-ureidopropyltrimethoxysilane) were studied and their effects on the properties of NBR composites were compared. As predicted, as the content of CNF in the elastomer matrix increased, the properties of the NBR/CNF composites improved. The application of silane coupling agents resulted in NBR/CNF composites with improved rheometric torque, crosslink density, electrical conductivity, and mechanical parameters, due to the enhanced compatibility and dispersion of the CNF in the NBR matrix. As the most promising results were obtained for APTS, chemical modification was also performed for this silane agent. Our comprehensive investigation revealed that the silanization of CNF with APTS contributed to improve dispersion and filler-polymer interactions, and as a consequence, enhanced the properties of the NBR/CNF composites:

- Rheometric measurements showed that application of APTS-silanized CNF resulted in the improvement of torque moment by approximately 53% and 12% compared to the raw NBR and to NBR filled with unmodified CNF, respectively.
- Swelling experiments indicated that the 10 APTS-CNF sample exhibited a very high crosslink density value (6.9 mol/cm^3^), which was 1.3 mol/cm^3^ higher than that measured for the composite filled with raw CNF.
- The tensile strength of the nitrile rubber composites was improved considerably, from 4.8 and 6.9 MPa (raw NBR and NBR/10CNF) up to 9.5 MPa for the composite with APTS-CNF, indicating the stronger reinforcing effect of CNF following the silanization process.
- SEM images showed better adhesion and dispersion of the silanized CNF in NBR matrix compared with unmodified CNF.
- NBR composites filled with APTS-CNF exhibited enhanced electrical conductivity (9.8 × 10^−12^ S) compared to pure NBR (3.4 × 10^−13^ S) and NBR filled with raw CNF (6.4 × 10^−12^ S), most likely due to the improved filler dispersion.
- Rheological experiments revealed improved polymer-filler interactions after the application of silane coupling agents, as evidenced by the reduced Payne effect.
- The flammability parameters of the 10 APTS-CNF composite were 20% lower compared to pure NBR, suggesting improved fire resistance in the case of the elastomer composites.

The results presented in this work show the great potential of silanization as an effective method for improving the reinforcing effects of CNF in nitrile rubber composites. This was evidenced by more satisfactory rheological, crosslink density, and mechanical results for NBR filled with APTS-CNF compared to those presented in our previous studies on NBR composites reinforced with the same loads of raw CNF or CNF with ionic liquids [17,25]. Carbon nanofibers modified with APTS in ethanol solution showed more reinforcing activity in the NBR composites than those applied in an in-situ process. On the other hand, in-situ silanization is rapid, does not require organic solvents, and gives only slightly less satisfactory results than the solvent method. Both presented techniques for CNF modification are relatively easy and time efficient, and show great potential for the versatile preparation of highly reinforced rubber composites for use in the production of cables, seals, and various shock-absorbing elements operating under high stresses.

## Figures and Tables

**Table 1:** Rheometric parameters obtained for CNF-filled compounds.

Sample Code	M_min_ (dNm)	ΔM (dNm)	τ_02_ (min)	τ_90_ (min)
Reference	0.41	5.10	6.04	28.06
5 CNF	0.52	5.72	3.89	24.92
10 CNF	0.64	6.99	3.06	27.47
15 CNF	0.65	7.89	2.91	27.46
10 O-CNF	0.50	6.80	3.10	27.55
10 APTS-CNF	0.59	7.81	3.06	27.47
10 CNF + APTS	0.60	5.92	3.98	27.80
10 CNF + MPTS	0.44	7.50	2.90	23.67
10 CNF + UPTS	0.51	7.63	2.61	21.69

M_min_—minimum torque moment; ΔM—torque increment; τ_02_—scorch time; τ_90_—curing time.

**Table 2:** Dynamic measurements data.

Sample Code	G′_min_ (Pa·10^5^)	G′_max_ (Pa·10^5^)	G″_max_ (Pa·10^4^)	ΔG′ (Pa·10^5^)
5 CNF	0.15	1.91	4.60	1.76
10 CNF	0.56	2.51	5.60	1.95
15 CNF	0.65	2.62	5.75	1.97
10 O-CNF	0.35	2.21	5.21	1.86
10 APTS-CNF	0.30	2.16	5.12	1.86
10 CNF + APTS	0.36	2.16	5.14	1.80
10 CNF + MPTS	0.31	2.09	5.01	1.78
10 CNF + UPTS	0.45	2.24	5.04	1.79

G′—storage modulus; G”—loss modulus; ΔG′—Payne effect.
